# Assessing the Immune Response of Atlantic Salmon (*Salmo salar*) after the Oral Intake of Alginate-Encapsulated *Piscirickettsia salmonis* Antigens

**DOI:** 10.3390/vaccines8030450

**Published:** 2020-08-11

**Authors:** Daniela Sotomayor-Gerding, José Miguel Troncoso, Alejandro Pino, Felipe Almendras, Mónica Rubilar Diaz

**Affiliations:** 1 Programa de Doctorado en Ciencias de Recursos Naturales, Universidad de La Frontera, Avenida Francisco Salazar 01145, Temuco 4811230, Chile; 2 Department of Chemical Engineering, Faculty of Engineering and Science, Universidad de La Frontera, Avenida Francisco Salazar 01145, Temuco 4811230, Chile; 3 Cargill Innovation Center, Camino a Pargua km 57, Colaco km 5, Calbuco, Chile; jose_troncoso@cargill.com; 4 Anasac Chile S.A., Veterinary Division, Almirante Pastene 300, Providencia 7500534, Santiago, Chile; alejandro.pino@tecnovax.com.ar; 5 SouthChile Ltda., Buenavista 16, Puerto Varas 5550000, Chile; Felipe.almendras@greenvolution.cl; 6 Scientific and Technological Bioresource Nucleus, BIOREN, Universidad de La Frontera, Avenida Francisco Salazar 01145, Temuco 4811230, Chile

**Keywords:** microencapsulation, alginate, aerodynamically assisted jetting system, *Piscirickettsia salmonis*, antigen, oral vaccine

## Abstract

Salmon rickettsial septicaemia (SRS) is the infectious disease that produces the highest losses in the Chilean salmon industry. As a new strategy for the control of SRS outbreaks, in this study we evaluated the effect of alginate-encapsulated *Piscirickettsia salmonis* antigens (AEPSA) incorporated in the feed as an oral vaccine to induce the immune response in Atlantic salmon (*Salmo salar*). Fish were distributed into three vaccination groups (injectable, oral high dose, oral low dose). Feed intake and fish growth were recorded during the trial. The *P. salmonis*-specific IgM levels in blood plasma were measured by ELISA. Alginate microparticles containing the antigen were effectively incorporated in fish feed to produce the oral vaccine. Incorporation of AEPSA did not affect the palatability of the feed or the fish appetite. Furthermore, the oral vaccine did not have a negative effect on fish growth. Finally, the oral vaccine (high and low dose) produced an acquired immune response (IgM) similar to the injectable vaccine, generating a statistically significant increase in the IgM levels at 840-degree days for both experimental groups. These findings suggest that AEPSA incorporated in the feed can be an effective alternative to boost the immune response in Atlantic salmon (*S. salar*).

## 1. Introduction

Salmon rickettsial septicaemia (SRS) is an infectious disease that affects salmon aquaculture around the world with a high economic impact [1]. SRS is responsible for 50% to 97% of the total disease-specific salmon mortality in the industry, accounting for annual direct and indirect loses between US$300–700 million [2]. The disease is caused by a non-motile obligate intracellular Gram-negative bacterium, *Piscirickettsia salmonis*, producing septicemia and high mortality in the fattening phase of the fish [3]. This pathogen was initially isolated from Coho salmon (*Oncorhynchus kisutch*) in Chile and has since also been reported in Canada, Ireland, Norway and Scotland [4].

Currently, vaccines and antibiotics are used to prevent and treat bacterial infections. However, the use of injectable vaccines requires considerable management and is also a stressful action for fish. On the other hand, the use of antibiotics has not delivered the expected protection, leading to their excessive use, which could eventually lead to the development of drug resistance in bacteria [5]. Furthermore, the probability of reporting mortalities due to SRS in any production cycle has been estimated in 82.5%, suggesting that neither the current use of vaccines or antibiotics against *P. salmonis* have been effective strategies to eliminate the infection [2]. Consequently, new technologies are needed to confront the disease.

One of the alternatives currently under study is the use of oral vaccines, offering significant advantages over needle-based vaccines, such as effortless application, improved safety, substantial reduction of stress as well as the possibility of the rapid vaccination of a large number of animals with reduced costs [6]. Furthermore, studies show that parenteral vaccination efficiently stimulates systemic responses but is a poor inducer of mucosal immunity, whereas oral administration of antigens results in stimulation of both systemic and mucosal responses [7]. Oral vaccines can be administered for primary vaccination or as a booster vaccine [8]. Its use as a booster is often preferred, as the induction of a robust secondary immune response, which is not generated when administered as a primary vaccine, has been demonstrated [9]. Oral vaccines have been tested for the control of infectious salmon anemia virus (ISAV) [10,11], infectious pancreatic necrosis virus (IPNV) [12,13] and SRS [5,14] in salmonids. Additionally, they have been used for the control of *Stretococcus iniae* in olive flounder [15], *Vibrio anguilarum* in turbot [16], *Aeromonas salmonicida* in goldfish [17] and *Aeromonas hydrophila* in Nile tilapia [18].

Nevertheless, the effectiveness of this approach is limited, since the vaccine can be degraded in the fish digestive system [19] and may also interact with feed components, not allowing the vaccine to reach the hindgut where antigens are absorbed [8]. Consequently, most antigens should be supplied in an encapsulated form. Polymers such as chitosan, Poly D, L-lactic-co-glycolic acid (PLGA), alginates, liposomes, and MicroMatrix^TM^ have been shown to be efficient for the oral delivery of vaccines in juvenile and older fish [20,21]. It has been shown that microencapsulated vaccines have several advantages for mucosal delivery. Microfold (M) cells (which are responsible for the transport of microbes and particles) are particularly accessible to microparticles and actively transport them into Peyer’s patches to initiate the immune response [22,23]. The presence of specialized antigen- sampling M-like cells has been proven in the fish intestine, together with the antigen-sampling ability of enterocytes and gut-associated macrophages [24,25]. Therefore, micro-encapsulation can be exploited to further enhance the uptake of inactivated bacteria or yeast and plant-derived vaccines. In this way vaccines that show suboptimal protection when delivered naked, possibly due to high intestinal antigen breakdown, can be improved with encapsulation [20].

Consequently, as a new strategy for the control of SRS outbreaks, the main objective of this study was to evaluate the effect of alginate-encapsulated *P. salmonis* antigens (AEPSA) incorporated in the feed as an oral vaccine to induce the immune response in Atlantic salmon. Several factors like nature of the antigen, dosage regimen and formulation of the vaccine influence the efficacy of oral vaccines [7]. Therefore, the effects of different antigen dose were evaluated and its effectiveness was compared with the injectable vaccine. A microencapsulation technique called the aerodynamically assisted jetting system was tested. This technique is capable of producing alginate capsules through ionic gelation, obtaining small particles of uniform shape and size, under simple and replicable conditions [26] in a process in which polymer solutions can be used without subjecting bioactive agents to high temperatures or any other extreme conditions [27]. Furthermore, this technique distinguishes as being economic, easily up-scalable and gentle with high throughput. These features make this process very well-suited for industrial application where effective large batch processes are favorable [28].

## 2. Materials and Methods

### 2.1. Ethics Statement

The experiment (internal study protocol ID number: CIC-COL 17.019) was reviewed by an internal animal welfare committee of the Cargill Innovation Center (Colaco, Calbuco, Chile) and executed under strict animal welfare standards following the Guidelines of the Canadian Council on Animal Care (CCAC) for the care and use of fish in research, teaching and testing.

### 2.2. Oral Vaccine Preparation

Oral vaccines were prepared by incorporating microparticles containing an injectable vaccine antigen (Providean Aquatec 1, Anasac, Chile) without excipients into the fish feed by vacuum coating. Two doses of the oral vaccine were prepared (See Table 1): (i) low dose: 30% of the recommended injectable dose (RID) to each fish for 10 days; (ii) high dose: 100% RID to each fish for 10 days, where the injectable dose recommended by the manufacturer corresponds to 0.1 mL (10^7^–10^8^ *P. salmonis* bacteria cells prior to inactivation by formaldehyde) per fish [29]. The antigenic solution without excipients used for oral vaccine preparation was 33.3× concentrated.

#### 2.2.1. Preparation of Alginate-Encapsulated *P. salmonis* Antigens (AEPSA)

Polymeric solutions (60 mL each) were prepared by mixing a volume (6.6 mL for low dose; 20 mL for high dose) of antigen solution (3.3 × 10^9^ bacteria/mL, *P. salmonis*-inactivated bacteria) with a previously prepared sodium alginate solution (36 mL, 5% *w*/*v*, Sigma Aldrich, St Louis, MO, USA). The resulting solution was adjusted with distilled water to give a total volume of 60 mL and 3% *w*/*v* alginate concentration (See Table 2).

Alginate microparticles were produced by ionic gelation using an aerodynamically assisted jetting system for the dispersion of microparticles [30]. The polymeric solution was pumped at 125 mL/h using a syringe pump (NE 8000, New Era Pump Systems Inc., Farmingdale, NY, USA), and the microparticles were stripped from the nozzle (XAPR 200A 303 SS, BETE Fog Nozzle, Inc, Greenfield, MA, USA) with the aid of a compressed air stream (1.25 bar), and dripped into a crosslinking solution (500 mL of CaCl_2_, 0.25 M, Sigma–Aldrich, St. Louis, MO, USA) from a height of 20 cm. The solution was kept under stirring at 300 rpm during the process. The formed microparticles (~16 g) were allowed to stand in the gelling bath for 30 min to ensure crosslinking between sodium alginate and calcium chloride. Microparticles were concentrated in a decantation funnel and kept in calcium chloride solution (~112 mL). The obtained microparticles were characterized (morphology and particle size) through optical microscopy (BX43, Olympus corporation, Tokyo, Japan) at 60× magnification. Two micrographs were obtained and ten microparticles from each picture were measured with the help of the QCapture Pro 7^TM^ software (QImaging, Surrey, BC, Canada).

#### 2.2.2. Preparation of Feed Pellets Infused with Antigen Microparticles

The generated microparticles (Section 2.2.1) in solution (128 g) were suspended in fish oil (672 g) and homogenized at 6000 rpm for 2 min with the aid of a digital Ultra TURRAX (T50, IKA^®^-Werke GmbH & CO., Staufen, Germany).

The final experimental feeds were produced through a vacuum coater (F-6 RVC, Forberg International AS, Oslo, Norway). The same nutritional level and raw material composition was used for each treatment. In the vacuum coating process, 4.2 kg of base pellet (Micro 50, EWOS, Chile) were added to the mixing chamber and then the suspended microencapsulated vaccine in fish oil (800 g) was added to the chamber. After hermetically closing the chamber, the ingredients were mixed, finally the vacuum coating process was started. In the process, the air was completely evacuated from the mixing chamber and also from the feed base pellet pores. After reaching 88 mbar, the air was released back into the chamber, pushing the oil into the empty pellet pores. In this way, the suitably-sized microparticles suspended in the fish oil were incorporated into the feed pellets.

With the purpose of detecting alginate microparticles inside the infused pellet, one fish feed pellet was observed in a scanning electron microscope (STEM SU-3500, Hitachi, Tokyo, Japan) using elemental mapping (QUANTAX EDS XFlash^®^ 6 detector, Bruker, Berlin, Germany) to detect the microparticles.

### 2.3. Experimental Design

The experiment was conducted at Cargill Innovation Center facilities in Colaco, Los Lagos region, Chile. All the fish in this study underwent a sanitary check, where a veterinarian certifies that the fish are disease-free before being incorporated into the trial, the presence of pathogens (SRS, Bacterial kidney disease (BKD), *Flavobacterium*, IPNV and *Piscine orthoreovirus* (PRV)) is analyzed by quantitative polymerase chain reaction (qPCR).

An acclimation period was carried out 2 weeks before the start of the experimental feeding. Environmental conditions for acclimation and trials were: 15 °C, pH 7.4, salinity 5 ‰, oxygen saturation 80–120%, a photoperiod of 24 light hours and water flow of 4.33 L/min.

**Figure 1:**
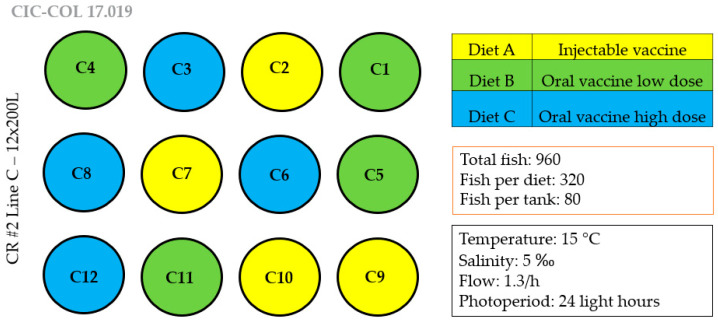
Experimental design for oral vaccine trial with alginate-encapsulated *Piscirickettsia salmonis* antigens infused in fish feed pellets. The experimental design included three fish groups (low dose, high dose, injectable vaccine) with four tanks per group and 80 fish per tank.

960 disease-free Atlantic salmon (*S. salar*) weighing on average 40.8 (±0.4) g each were distributed into 12 circular 200 L tanks containing fresh water at a density of 16 kg/m^3^. The experimental design (Figure 1) included three fish groups (4 tanks per group; 80 fish per tank). The groups were: (a) control: fish vaccinated with injectable vaccine; (b) oral vaccine high dose: fish fed with microencapsulated oral vaccine added in feed for 10 days; (c) oral vaccine low dose: fish fed with microencapsulated oral vaccine added in feed for 10 days. Fish were fed at 1–2% of their body weight per day.

### 2.4. Feed Intake and Fish Growth Assessment

Unconsumed feed was collected during the trial to calculate feed intake. Uneaten pellets were spilled out of the tanks within 10 min post-feeding and filtered off from the outlet water using an automatic collection system. Residual pellets were removed from the filters and kept in a drying cabinet for 24 h at 70 °C. The amount of consumed feed was calculated as the difference between the dry weight of the feed used and the dry weight of unconsumed feed. The feed intake (%) was calculated as follows: Feed intake (%) = (Consumed feed (g)/Supplied feed (g)) × 100.

The fish weight and length were recorded during the trial; eight fish per tank were taken at four sampling points (0 degree days after vaccination (DD), 300 DD, 600 DD and 840 DD). These same fish were used to determine IgM levels in blood plasma (Section 2.5). The dietary effects on growth were determined by evaluating weight gain, length gain and specific growth rate (SGR). The indices were calculated as follows: weight gain (g) = final weight − initial weight; length gain (cm) = final length − initial length; SGR, specific growth rate (%/day) = 100 × (ln final weight (g) − ln initial weight (g))/time (days) [31].

### 2.5. Effect of the Experimental Feed on the Immune System

Eight fish from each tank were taken at four sampling points (basal, 300 DD, 600 DD and 840 DD) and euthanized by an overdose of benzocaine (BZ^®^-20, Veterquimica, Chile). Blood samples were taken from the caudal vein with 1 mL heparinized syringe, centrifuged at 5000 *g* for 10 min and stored at −20 °C until use. The *P. salmonis*-specific IgM levels in blood plasma were measured by ELISA (Laboratory of Immunology and stress of Aquatic Organisms, Instituto de Patología Animal, Universidad Austral de Chile, Valdivia, Chile). 

The ELISA assay was performed as described by Birkbeck, et al. [32], with some modifications. Firstly, Nunc-Immuno 96 MicroWell solid plates (Sigma, St. Louis, MO, USA) were seeded with 1 mg of total protein extracts (10 ng/µL, diluted in 0.05 M carbonate buffer, pH 9.6) from *P. salmonis* strain LF-89, for 12 h at 4 °C. Later, unbound antigens were removed from the wells washing three times with phosphate buffered saline (PBS) containing 0.05% Tween 20 (PBS-T) and then blocked with 3% bovine serum albumin (BSA) in PBS-T (blocking solution) for 1 h at 18 °C; after that, 100 µL of serum samples were added to the wells and incubated for 2 h at 18 °C, then washed three times with PBS-T. For the anti-*P. salmonis* IgM detection, the plates were incubated with monoclonal anti-salmon IgM antibody (IgG clone 3H7/E1, ANGO, San Ramon, CA, USA) diluted 1:1000 in blocking solution at 18 °C for 1 h. After twice washing with PBS-T, 100 mL of an anti-mouse antibody conjugated to horseradish peroxidase (Thermo Fisher Scientific, Waltham, MA, USA) were added (diluted 1:2500 in PBS-T) followed by incubation for 2 h at 25 °C. Finally, antigen-antibody complex was revealed using 50 µL per well of TMB (TetraMetilBencidin, 1 mg/mL in dimethylsulfoxide) during 10 min. The reaction was stopped by addition of 50 µL of 1.8 M H_2_SO_4_ and the absorbance value for each sample was measured at 450 nm in a 96-well plate reader (Synergy^TM^ 2 Multi- Detection Microplate Reader, Biotek Instruments, Inc., Winooski, VT, USA) with the Gen5^TM^ v1.10 software. The color intensity in the well was proportional to the anti-*P. salmonis* salmon IgM concentration in the sample. All samples were analyzed in triplicate, performing pools of two fish serum with their respective positive, negative and blank controls.

### 2.6. Statistical Analysis

At least three determinations were made for all assays and results were expressed as the mean and standard deviation. Results of this study were subjected to a one-way analysis of variance (ANOVA). Significant differences (*p* ≤ 0.05) between means were determined by Tukey’s tests. Non-parametric Kruskal–Wallis test was applied to evaluate differences in fish length.

## 3. Results

### 3.1. Characterization of Microparticles and Incorporation in Fish Feed Pellets

Microencapsulation of *P. salmonis* antigens in alginate using the aerodynamically assisted jetting system was evaluated. An amount of approximately 16 g of alginate antigen microparticles (humid mass) were produced after dispersing 60 mL of polymeric solution. Microparticles were characterized, morphology and particle size, through optical microscopy. The aerodynamically assisted jetting system proved to be effective in preparing alginate-antigen microparticles with a small particle size (<20 μm). 

**Figure 2:**
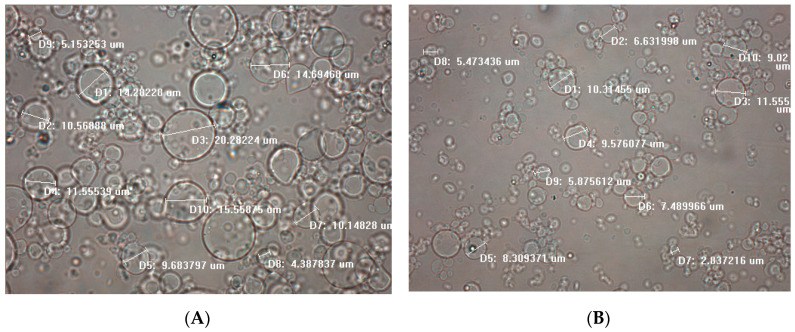
Optical microscopy (60×) of alginate encapsulated *Piscirickettsia salmonis* antigens. (**A**) picture 1; (**B**) picture 2: ten microparticles were measured in each micrograph with the help of the QCapture Pro 7^TM^ software.

Microparticles observed in picture 1 (Figure 2A) had a mean particle size of 11.63 ± 4.81 µm, and microparticles with diameters ranging from 4.39 and 20.28 µm were observed, while the microparticles in picture 2 (Figure 2B) had an average size of 7.71 ± 2.59 µm, particles with a maximum size of 11.56 µm and a minimum of 2.84 µm were observed. Most microparticles obtained had a soft round shape (Figure 2).

**Figure 3:**
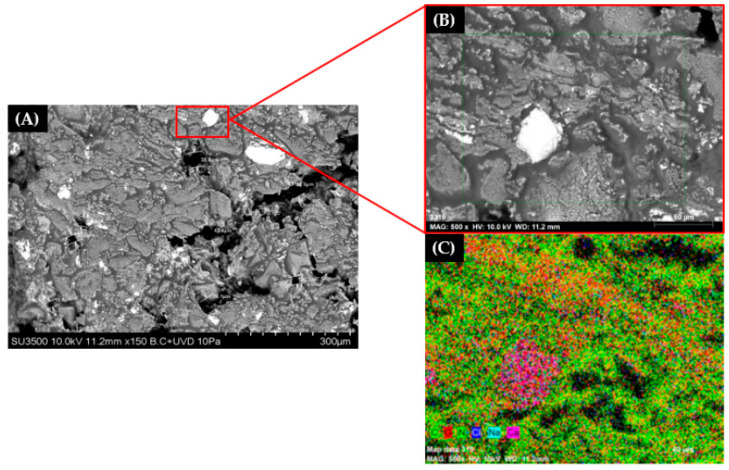
Scanning electron microscopy of a fish feed pellet infused with antigen alginate microparticles. (**A**) original micrograph at 150×; (**B**) 500× magnification; (**C**) elemental mapping at 500× magnification.

Alginate microparticles were dispersed in fish oil and the solution was homogenized before incorporation in fish feed pellets by the vacuum infusion process. An oil concentration of 16% *w*/*w* was selected to ensure total incorporation of the bioactive into the pellets. One fish feed pellet infused with alginate microparticles was observed in a scanning electron microscope (Figure 3) to confirm microparticle incorporation. Microparticles with a form and size similar to the obtained were observed in the picture. Increasing the magnification, a particle size smaller than 50 µm was confirmed (Figure 3B). Furthermore, using elemental mapping it was confirmed that these points were associated with calcium (Figure 3C), as expected for these microparticles after crosslinking with calcium chloride.

### 3.2. Acceptability of the Experimental Feed and Its Effect on the Fish Growth

The fish were fed with the microencapsulated oral vaccine added in feed, one group in low dose and another in high dose, while a third group was used as a positive control, being vaccinated by injection. After the experimental period of ten days, the fish were fed with commercial feed. The feed intake was monitored to evaluate the acceptability of the experimental oral vaccine. During experimental feeding with the oral vaccine, the fish consumed all the feed delivered, both low-dose and high-dose groups. In addition, intake rates were monitored throughout the trial to assess whether vaccination could have an effect on fish appetite. Unconsumed feed was less than 10% for all groups. The lowest feed intake rate was obtained in the fish vaccinated by injection with 90.81%, followed by the high-dose oral vaccine with 94.02% and lastly, the low-dose oral vaccine with 95.23%. The analysis of variance determined that there were no differences on the feed intake throughout the trial among the experimental groups (*p*-value: 0.16) (Table 3).

**Figure 4:**
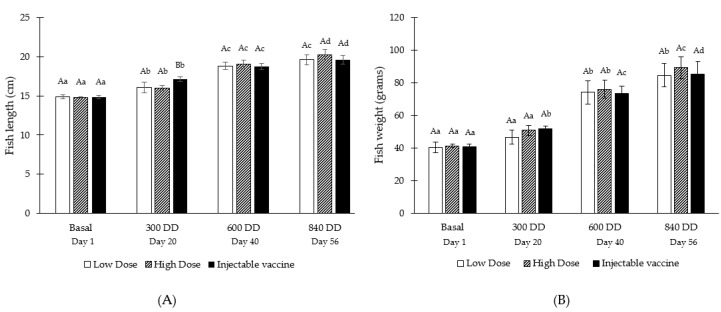
Fish length (**A**) and weight (**B**) of Atlantic salmon (*Salmo salar)* fed with experimental diets at different sampling points during the trial. Different capital letters indicate significant differences (*p* ≤ 0.05) of the length or weight among the different experimental groups (low dose, high dose, injectable vaccine) within the same sampling point. Different lowercase letters indicate significant differences (*p* ≤ 0.05) of the length or weight among the different sampling points (baseline, 300, 600 and 840 DD) within the same experimental group.

The effects of microencapsulated oral vaccine consumption on fish growth were evaluated to determine if the experimental diet could interfere with nutrient absorption. Figure 4 shows the fish weight and length at four sampling points (basal, 300, 600 and 840 DD) for the three experimental groups (low dose, high dose, injectable vaccine). 

When comparing the average height of the experimental groups within the same sampling point, at 300 DD it is observed that the group vaccinated via injection has a significantly larger size than the other groups (Figure 4A). However, this effect is not observed in the other comparisons, where no significant differences in height are found when comparing among the experimental groups. As expected, a significant increase in the average size of fish occurs between different sampling points. Only at 840 DD the low-dose oral vaccine did not have a significant change in size between 600 and 840 DD (Figure 4A).

Evaluating the weight, there were no significant differences between the experimental groups within the same sampling point. Assessing the weight of the fish at different sampling points, for the groups of oral vaccine in low dose and high dose there were no significant differences between 0 and 300 DD, however, for the group vaccinated via injection, a significant increase was observed. Between 300 and 600 DD a significant increase in weight is observed for all groups and between 600 and 840 DD a significant increase is observed for the groups vaccinated by oral route in high dose and those vaccinated by injectable route (Figure 4B).

Weight gain, length gain and specific growth rate (SGR) of Atlantic salmon (*S. salar*) fed with experimental diets were determined to further evaluate fish growth (Table 4). Growth rates for the experimental groups of low-dose oral vaccine and injectable vaccine were very similar. The weight gain was 44.12 and 44.2 g for the low dose oral vaccine and for the injectable vaccine, respectively. The length gain was 4.77 cm for the low dose oral vaccine and 4.78 cm and for the injectable vaccine. Finally, the SGR was 1.37 for the low dose oral vaccine and 1.35 for the injectable vaccine. The highest growth rate was obtained for the high-dose oral vaccine, with a weight increase of 47.93 g, a height increase of 5.48 cm and an SGR of 1.42. However, statistical analysis showed no significant differences (95% confidence) among the different experimental groups (low dose, high dose, injectable vaccine).

### 3.3. Effect of the Oral Vaccine on the Immune Response of Atlantic salmon

Finally, the effect of the oral vaccine on the immune response of the fish was evaluated by analyzing serum IgM levels, evaluating two doses of the oral vaccine and comparing them with the immune response generated by the commercial injectable vaccine. To ensure that the immune response detected corresponds to the vaccine and not a response to a disease, all the fish in this study went through a sanitary check, which confirmed that all fish were disease free at the start of the trial; furthermore, the analysis was performed for a *P. salmonis*-specific IgM.

**Figure 5:**
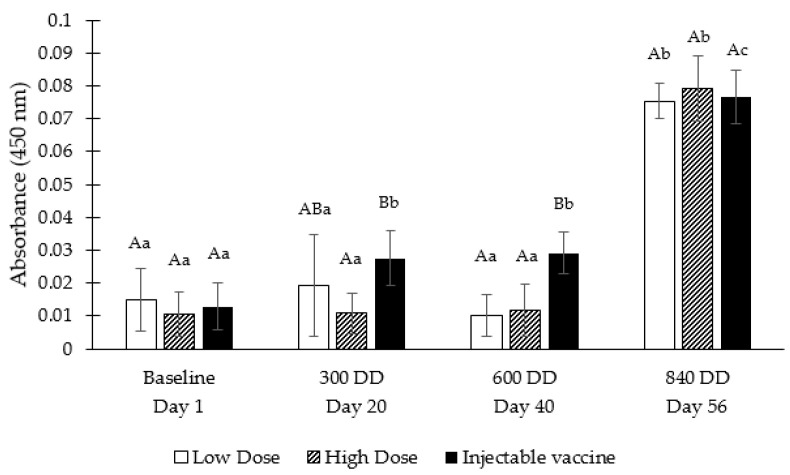
Serum *Piscirickettsia salmonis*-specific IgM levels of Atlantic salmon vaccinated with: oral vaccine low dose, oral vaccine high dose and injectable vaccine. Different capital letters indicate significant differences (*p* ≤ 0.05) of the absorbance among the different experimental groups (low dose, high dose, injectable vaccine) within the same sampling point. Different lowercase letters indicate significant differences (*p* ≤ 0.05) of the absorbance among the different sampling points (baseline, 300, 600 and 840 DD) within the same experimental group.

Figure 5 shows the absorbance at 450 nm (which was proportional to the anti-*P. salmonis* salmon IgM concentration in the sample) of Atlantic salmon serum samples after applying the ELISA protocol for the three experimental groups (low dose, high dose, injectable vaccine) at four sampling points (basal, 300, 600 and 840 DD).

Comparing IgM levels between groups within the same sampling point, significant differences were observed among the types of vaccines at 300 and 600 DD: at 300 DD, the low-dose oral vaccine had significantly lower IgM levels than the injectable vaccine; at 600 DD, the group vaccinated via injection had significantly higher IgM levels than the oral vaccine in both low and high doses. However, at 840 DD, no significant differences were observed among the groups.

When comparing IgM levels for the same group at different sampling points, the oral vaccines both in low and high doses did not have significant changes between 0 and 600 DD, only at 840 DD a significant increase in levels of IgM was observed, increasing 7-fold for the low-dose oral vaccine and 6.8-fold for the high-dose oral vaccine when comparing with the previous sampling point. In the case of the injectable vaccine, the IgM levels were gradually increasing, with significant differences being observed between 0 and 300 DD, and then, between 600 and 840 DD another significant increase occurs, increasing 2.6-times when compared with the previous sampling point.

In brief, a significant increase in the IgM levels was observed for all groups at 840 DD, thus confirming that the oral vaccine (both in the high and low doses) can increase the immune response of the fish. Furthermore, when comparing IgM levels at 840 DD among the groups, no significant differences were detected, indicating that the oral vaccine could reach IgM levels similar to those obtained with the injectable vaccine (Figure 5).

## 4. Discussion

The use of oral vaccines is one of the alternatives under study to support the control of bacterial and viral diseases in salmonids and other aquaculture species [20]. In this study, the effect of alginate-encapsulated *P. salmonis* antigens (AEPSA) incorporated in the feed as an oral vaccine to induce the immune response in Atlantic salmon were evaluated. The effects of different antigen dose were evaluated and the oral vaccine effectiveness was compared with the commercial injectable vaccine. The *P. salmonis* antigen was supplied in a microencapsulated form, to prevent high intestinal breakdown and enhance uptake.

Currently there are a wide variety of techniques and polymers used for microencapsulation, which are selected according to the application and expected characteristics of the microparticle [33]. Among the polymers that have been tested in oral vaccines for fish with good results, there are: chitosan, Poly D,L-lactic-co-glycolic acid (PLGA), alginates, liposomes, and MicroMatrix^TM^ [21]. Studies suggest that alginates might be particularly suitable to deliver antigens at mucosal surfaces in fish, showing their ability to protect antigens while passing through the digestive tract, and to diffuse through the gut mucus layer, thereby reaching the enterocyte surface. Once in contact with the epithelium, alginates might be actively taken up by antigen-sampling cells [20]. The alginate polymer has been used for the encapsulation of bacterial pathogens that affect salmonids such as *Vibrio harveyi* [34], *Flavobacterium psychrophilum* [35], *Lactococcus garviae* [36], in addition to some viruses such as Infectious haematopoietic necrosis virus (IHNV) [37] and IPNV [9,38].

In this study, *P. salmonis* antigens were effectively encapsulated in alginate using the dispersion technique known as aerodynamically assisted jetting system. In recent years, this technology has been effectively tested for handling a wide range of advanced materials spanning structural, functional and more importantly biological (from cells to whole organisms) materials [27] and is currently under study for biomedical applications [39,40,41]. This technology can accomplish an encapsulation efficiency between 78% and 97% depending on the compound used to generate the crosslinking, as reported in a study on the encapsulation of IPNV antigens that were incorporated into an oral vaccine for Atlantic Salmon [12,42].

In general, alginate microparticles can be found in a wide range of sizes (0.1–1000 µm) [43] and have a negative zeta potential close to −25 mV [44,45]. In this case, the aerodynamically assisted jetting system allows to adjust parameters such as flow rate or air pressure to reach the expected particle size [27]. A small particle size was achieved, obtaining diameters ranging from 2.84 to 20.28 µm. Since the antigen used in this study corresponds to *P. salmonis* bacteria inactivated by formaldehyde, which size ranges from 0.1 to 1.8 µm in diameter [46], an effective immobilization of the antigen in the microparticle is expected.

A small particle size is also sought because these microparticles must be incorporated in the porous matrix of the pellet. Draganovic et al. [47] reported that most pore sizes in the fish feed pellet (d = 8.7 mm) were below 330 µm; therefore, we expect these microparticles, with a mean particle size lower than 20 µm, to be easily incorporated in the pellet through the vacuum infusion coating method. Furthermore, incorporation was confirmed after analysis by SEM-EDS where particles with a size smaller than 50 µm were found inside the pellet and elemental mapping showed that these particles were associated to calcium, as expected for calcium-alginate microparticles since the external gelation process occurs with the diffusion of the calcium ion from a source surrounding the hydrocolloid to the neutral pH alginate solution [48]. The incorporation of microparticles in pellets for the development of oral vaccines is a technique that has already been proven in several studies. For example, the method has been used for the incorporation in pellets of antigens against IPNV [13], *S. iniae* [12] and *P. salmonis* [11] with effective results.

The experimental feed had a good acceptability, the fish consumed all the oral vaccine supplied, in both low and high doses, indicating that the addition of AEPSA did not affect the palatability of the fish feed. In addition to evaluating oral vaccine consumption, feed intake rates (from non-experimental feed) were assessed after vaccination throughout the trial to assess whether it had an effect on fish appetite. The feed intake throughout the trial was higher for the oral vaccine in the low dose, followed by the oral vaccine in the high dose and finally by the fish vaccinated by injectable route. It could be inferred that the addition of the antigen in higher doses would decrease the appetite of the fish in the long term. However, no significant differences were obtained between the groups and the orally vaccinated groups had higher intake rates than the injectable vaccine groups. Consequently, it cannot be concluded that the oral vaccine had a negative effect on fish appetite.

Evaluating changes in weight and size of the fish over time, some differences were observed between the groups, for example, at 300 DD the group vaccinated via injection had a significantly larger size than the other groups, and between 600 and 840 DD a significant increase in weight was observed for the groups vaccinated by oral route in high dose and those vaccinated by injectable route but not for the orally vaccinated with the low dose. However, when evaluating the weight gain, height gain and the specific growth rate during the trial (between 0 and 840 DD), no significant differences were observed between the groups, therefore, the different experimental diets do not have a negative effect on fish growth.

A successful oral vaccination must not affect gut function in order to maintain the assimilation rate in the intestine of vaccinated fish. In our study, results suggest that administration of the oral vaccine induces no effect in nutrient assimilation. Alginate encapsulated antigens have been evaluated in oral vaccines in previous studies for the control of IPNV in Atlantic salmon [42], *V. anguilarum* in rainbow trout [49], *A. salmonicida* in goldfish [17] and *A. hydrophila* in Nile tilapia [18], without showing negative effects on the palatability of the food or on the nutrient absorption capacity of the fish. Therefore, our results agree with these previous studies.

Finally, it was confirmed that AEPSA incorporated into the feed as an oral vaccine can induce the immune response on Atlantic salmon, generating a significant increase in the IgM levels of the fish. IgM is the major systemic antibody in teleost fish [50]; therefore, it was used as an indicator of the immune response in this study. The injectable vaccine elicits a strong and prolonged immune response, where the onset of immune competition occurs at approximately 600 DD after vaccination [14,29]. In this study, a gradual increase in the immune response was observed, obtaining the highest increase between 600 and 840 DD. For the oral vaccine, previous studies indicate that it can induce an immune response characterized by a sudden increase in the specific antibody in the blood [49,51]. This effect can be observed in our study, where IgM levels increased 7-fold for the low-dose oral vaccine and 6.8-fold for the high-dose oral vaccine between 600 and 840 DD. Similarly, in an oral vaccine prepared incorporating an SRS antigen into the Micromatrix^TM^ oral delivery system, the highest titers of systemic anti-*P. salmonis* antibodies were obtained between 600 and 900 DD [14].

It should be considered that the doses of antigen contained in the oral vaccine are much higher than those delivered by the injectable route. The high dose oral vaccine was formulated to deliver 10-times the dose contained in an injectable vaccine per fish based on previous reports [14], whereas the low dose oral vaccine was formulated to deliver 3-times the dose of an injectable vaccine. The highest number of doses in the oral vaccine was added considering that there would be losses of the antigen in the production processes of the oral vaccine, possible losses during the storage of the feed and possible losses caused by the passage of the feed through the gastrointestinal tract of the fish [19]. The results of this study suggest that immune response levels similar to those of the injectable vaccine could be achieved using the low dose oral vaccine, with only 3-times the recommended injectable dose, and an increase of the dose to 10-times would be unnecessary, since no significant differences were obtained when comparing the IgM levels of the low dose with those of the high dose. This effect can be generated by the phenomenon known as oral tolerance, which is defined as the hypo-responsiveness to orally administered antigens [7,52]. In mammals, high doses of ingested antigens will induce anergy (inability of lymphocytes to react to the presence of an antigen) and deletion of antigen-specific T cell whereas low antigen dose will rather trigger the development of regulatory T cells [20]. In fish, although the mechanisms of tolerance induction have not been fully understood; antigen dose and route of administration have been associated to oral tolerance [17,49] being for the most part illustrated by decreased antibody response following repeated antigen exposure [7].

This study provides a precedent on the use of alginate microparticles as a delivery system for *P. salmonis* antigens. However, to generate an effective oral vaccination strategy, future studies should focus on optimizing the formulation, determining the losses of the antigen in the production process of the oral vaccine, in its storage, supply, as well as in the process of digestion. Furthermore, the mechanisms of immune response generation after oral vaccination, as well as the factors that induce tolerance, are still not completely clear, therefore further research is needed in this area.

## 5. Conclusions

Our study demonstrates that *P. salmonis* antigens can be microencapsulated in alginate using an aerodynamically assisted jetting system, generating small microparticles that can be incorporated into fish feed pellets and produce an oral vaccine. It was confirmed that the addition of AEPSA does not induce effects on the palatability of the feed and that oral vaccination does not affect the appetite of the fish. Furthermore, feeding with the oral vaccine did not have a negative effect on fish growth.

The experimental oral vaccine effectively enhanced the immune response of fish, achieving levels of IgM similar to those obtained from the injectable vaccine. These findings suggest that alginate-encapsulated *P. salmonis* antigens incorporated into the feed can be an effective alternative to enhance the immune response in Atlantic salmon.

## Figures and Tables

**Table 1:** Characteristics of the experimental oral vaccines prepared with alginate encapsulated *Piscirickettsia salmonis* antigens in high dose and low dose.

Oral Vaccine	Total Doses/Fish	% of the RID/Fish	Dose/ Gram of Feed	Bacteria Cells/ Gram of Feed	Grams of Feed/ Day/Fish
Low dose	3	30%	0.44	4.4 × 10^7^	0.68
High dose	10	100%	1.33	1.33 × 10^8^	0.75

**Table 2:** Characteristics of polymeric solutions used to prepare experimental oral vaccines containing alginate-encapsulated *Piscirickettsia salmonis* antigens (AEPSA).

Oral Vaccine	Number of Doses for 5 kg of Feed	Antigen Volume (mL)	Alginate Solution (mL)	Distilled Water (mL)	Total Polymeric Solution (mL)
Low dose	2206	6.6	36	17.4	60
High dose	6667	20	36	4	60

**Table 3:** Feed intake rates for experimental diets (low dose oral vaccine, high dose oral vaccine, injectable vaccine) during the vaccination period and throughout the trial.

Group	Feed Intake (%) during Vaccination Period	Feed Intake (%) during the Entire Trial
Low dose	100 a	95.23 ± 1.83 a
High dose	100 a	94.02 ± 3.87 a
Injectable vaccine	100 a	90.81 ± 2.93 a

Different lowercase letters in a column indicate significant differences (*p* ≤ 0.05) of the feed intake among the different experimental groups (low dose, high dose, injectable vaccine).

**Table 4:** Weight gain, length gain and specific growth rate (SGR) of Atlantic salmon (*Salmo salar*) fed with experimental diets (low dose oral vaccine, high dose oral vaccine, injectable vaccine).

Group	Weight Gain (grams)	Length Gain (cm)	SGR (%/day)
Low dose	44.12 ± 5.59 a	4.77 ± 0.36 a	1.37 ± 0.12 a
High dose	47.93 ± 7.40 a	5.48 ± 0.73 a	1.42 ± 0.17 a
Injectable vaccine	44.20 ± 8.45 a	4.78 ± 0.77 a	1. 35 ± 0.20 a
*p*-value	0.706	0.219 ^1^	0.816

Different lowercase letters in a column indicate significant differences (*p* ≤ 0.05) among the different experimental groups (low dose, high dose, injectable vaccine). ^1^ Non-parametric Kruskal–Wallis test was applied to evaluate differences in length gain.
